# Resiliency in Persons Experiencing Homelessness: A Concept Analysis Using the Evolutionary Framework

**DOI:** 10.1111/jan.16440

**Published:** 2024-09-10

**Authors:** Christian Ketel, Samereh Abdoli

**Affiliations:** ^1^ Vanderbilt University School of Nursing Nashville Tennessee USA; ^2^ University of Tennessee College of Nursing Knoxville Tennessee USA

**Keywords:** homelessness, resiliency, Rogers' evolutionary method, unhoused

## Abstract

**Introduction:**

Homelessness is a critical societal issue, with those affected facing disproportionate chronic and acute health conditions. At the core of understanding their experiences is the concept of resiliency. Understanding resiliency in homelessness is crucial because it highlights the dynamic interplay between inherent qualities and external challenges, underscoring the need to reassess societal value judgements and inform nursing practices in research, education and advocacy.

**Aim:**

This paper aims to conduct a comprehensive concept analysis to propose a revised definition of resiliency in the context of homelessness.

**Methods:**

Rogers's evolutionary method was the analytical tool of choice, perfect for examining the fluid and evolving nature of resiliency within the context of homelessness. The method entails analysing the concept's transformation over time and across disciplines, accepting variability in definitions based on the chronological and contextual constructs.

**Discussion:**

The findings revealed that resiliency in homelessness is an inherent quality and a developed response shaped by the interplay of innate capacities and environmental challenges. It uncovered a need to reassess societal value judgements. Resiliency among people without housing suggests strengths that go unrecognised by conventional measures.

**Conclusion:**

The analysis concludes that resiliency is not a static trait but a dynamic process encompassing individual, social and environmental dimensions. In each case, nursing is poised to make a significant difference in nursing research, practice, education and advocacy, potentially inspiring new approaches and interventions. No public or private was used in the development of this article.

## 1 Introduction

Homelessness is a complex and multifaceted societal issue faced by many countries, including the United States (Funk et al. 2022). Those who experience homelessness suffer significantly more from chronic and acute healthcare conditions and are some of the most vulnerable in our society (Onapa et al. 2022). Persons and families experiencing homelessness face various challenges, ranging from the immediate concerns of securing food and shelter to the long‐term psychological and social impacts of living without a permanent home (Mejia‐Lancheros et al. 2021).

Central to understanding the experiences of unhoused individuals is the concept of resiliency. However, there is no consensus on a firm definition (Liu et al. 2020). Understanding the concept of resiliency is crucial to understanding homelessness because it highlights the capacity of individuals to adapt and survive despite severe adversity, clarifying the complex personal and social challenges they face. Recognising resilience also helps develop more effective and empathetic support systems and policies that acknowledge the strengths and potential of those experiencing homelessness.

Resiliency emerges as a vital concept in the context of homelessness. The continuous exposure to diverse stressors, including environmental, social and physical, necessitates resiliency for this vulnerable population to navigate their daily lives effectively (Nilsson, Nordentoft, and Hjorthøj 2019). However, the definition and parameters of resiliency, especially in the context of adult homelessness, remain enigmatic, fluid and diverse across various studies and disciplines.

In contemporary literature, the concept of resiliency has evolved considerably. It was initially used as a descriptive term to define a physical material's property to retain physical integrity after applied stress (Mayar, Carmichael, and Shen 2022). In a reverse kind of personification, resiliency was found to have a parallel application in health sciences. Initially proposed in individual psychology, the term resiliency was first used outside of manufacturing as a core component of a person's ego to maintain psychological integrity after acute or prolonged psychological, situational or environmental stress (Tryon and Radzin 1972). The term resiliency has also found applications associated with communities' or society's ability to rebound after extreme natural and man‐made disasters (Tan et al. 2023). In this early research stream, researchers often used the term resiliency to describe children who thrived despite adverse environments (Crittenden 1985; Wyman et al. 1992; Leonard 1991). For instance, researchers Cadell, Karabanow and Sanchez (2001) define resiliency in their exploration of homeless youth as ‘the ability to adapt to, cope with, and even be strengthened by adverse circumstances (p. 26)’. This definition aligns with the perspective that resiliency is an immutable static trait, but the concept of resiliency has evolved.

Over time, the concept of resiliency has expanded to encompass all ages and various aspects of life, such as the internal processes of individuals, their external support systems and the intersectionality of their identities (Han et al. 2023; Tippens et al. 2023). In the realm of homelessness, resiliency is often observed not only as a trait but also as a process wherein individuals tap into their inner strengths and available resources to overcome the challenges of living without stable housing (Karadzhov, Yuan, and Bond 2020; Grattan et al. 2022).

### 1.1 Purpose and Aim

This paper describes the concept of resilience in adult homeless populations, offering a foundation for further nursing research, practice, education and advocacy. This review will support a conceptual analysis and definition of resiliency in people experiencing homelessness, providing insights into resilience beyond traditional views and emphasising human endurance and adaptation amid homelessness.

## 2 Method

The phenomenon of homelessness presents a complex tapestry of challenges, both for those experiencing it and for the professionals striving to address it. Resiliency is central to understanding and mitigating these challenges—a multifaceted and dynamic quality that plays a critical role in the lives of the unhoused. This study adopts a structured and evolutionary approach to concept analysis to delve deeper into this concept and its implications within the realm of homelessness. Through Rogers' evolutionary method, this research aims to dissect and contemporise the understanding of resiliency, thereby shedding light on its significance and application in this context. This introduction serves as a prelude to a detailed exploration of the framework and methodology employed in the study, setting the stage for a nuanced examination of resiliency among the homeless population.

### 2.1 The Framework: Evolutionary Method in Action

Rogers's evolutionary method of concept analysis was used to scrutinise and contemporise the concept of resiliency within the context of homelessness. This analytical framework facilitates examining the concept's definition and comprehension as it has transformed through time and across various academic disciplines (Rodgers and Knafl 2000). Such an approach is essential to crystallise a nuanced understanding of resiliency congruent with the authentic experiences of unhoused individuals and the healthcare professionals dedicated to their care.

The evolutionary method provides a holistic framework for the analysis of concepts, underscoring the natural evolution and progressive refinement of concepts over time within different contexts (Rodgers and Knafl 2000). It acknowledges the fluid and evolving nature of concepts, which are subject to change per the progression of research, cultural dynamics and societal advancements. This method accepts the variability of concept definitions based on the chronological period of study, the academic discipline and the context in which the concept is examined (Rodgers and Knafl 2000).

Adopting Rogers's evolutionary method entails initially selecting a concept that bears significance within the researcher's domain. The subsequent steps include articulating the purpose of the analysis and delineating its scope, considering specified timeframes, disciplines and contexts. A comprehensive interdisciplinary literature review is conducted, drawing on various academic sources. This literature is then meticulously scrutinised to ascertain the concept's definitions, descriptions and applications, emphasising identifying commonalities, disparities and evolutionary trends (Rodgers and Knafl 2000).

This phase also involves the identification of surrogate terms and related concepts. The defining characteristics of the concept are then distilled based on consistent attributes identified throughout the literature. A model case is constructed to enhance understanding and provide a concrete exemplification of the idea, utilising the defining qualities. The method further requires identifying antecedents and consequences—conditions that precede the concept and the outcomes that arise. Finally, the researcher solidifies empirical referents—observable indicators of the concept's existence in actual or hypothetical scenarios (Rodgers and Knafl 2000).

Applying this method to the concept of resiliency aims to illuminate the homeless population's intrinsic adaptive capacities and coping strategies. It will also emphasise the critical importance of nurturing resilience through avenues of nursing research, policy formulation and intervention development. This paper aims to synthesise the heterogeneous scholarly perspectives around resiliency, thereby providing a comprehensive portrayal of resiliency as exhibited by individuals navigating the experience of homelessness (see Table 1).

**TABLE 1:** Application of Rogers' evolutionary method to resiliency in homelessness.

Step in Rogers' evolutionary component	Application to resiliency in homelessness	Key findings and examples
1. Concept selection	Identified ‘resiliency’ as a critical concept within homelessness research	Resiliency is essential for understanding how individuals navigate and overcome the challenges of homelessness
2. Purpose of analysis	Aimed to redefine resiliency in the context of homelessness to inform nursing practice and policy	Highlighted the need for a dynamic definition encompassing inherent qualities and external influences
3. Identify uses of concept	Conducted a comprehensive literature review to explore various definitions and applications of resiliency	Found resiliency is an inherent trait and a developed response to adversity
4. Determine attributes	Analysed literature to identify key attributes of resiliency in homelessness	Identified attributes: adaptability, problem‐solving ability, social support and hope
5. Identify antecedents	Examined conditions that precede the development of resiliency	Found that psychological foundation, social networks and environmental factors are critical antecedents
6. Identify consequences	Explored outcomes that result from resiliency	Consequences include elevated self‐awareness, emotional exhaustion, profound interpersonal bonding and potential dependency on specific environments
7. Model Case	Created a hypothetical case study to illustrate the concept	The case study of ‘Jane’ demonstrated how resiliency manifests through coping strategies, adaptability and social support
8. Empirical Referents	Identified observable indicators of resiliency in real‐world scenarios	Indicators include resourcefulness, emotional stability and forming supportive social networks

*Note:* Rodgers and Knafl (2000).

### 2.2 Review of the Literature

**FIGURE 1:**
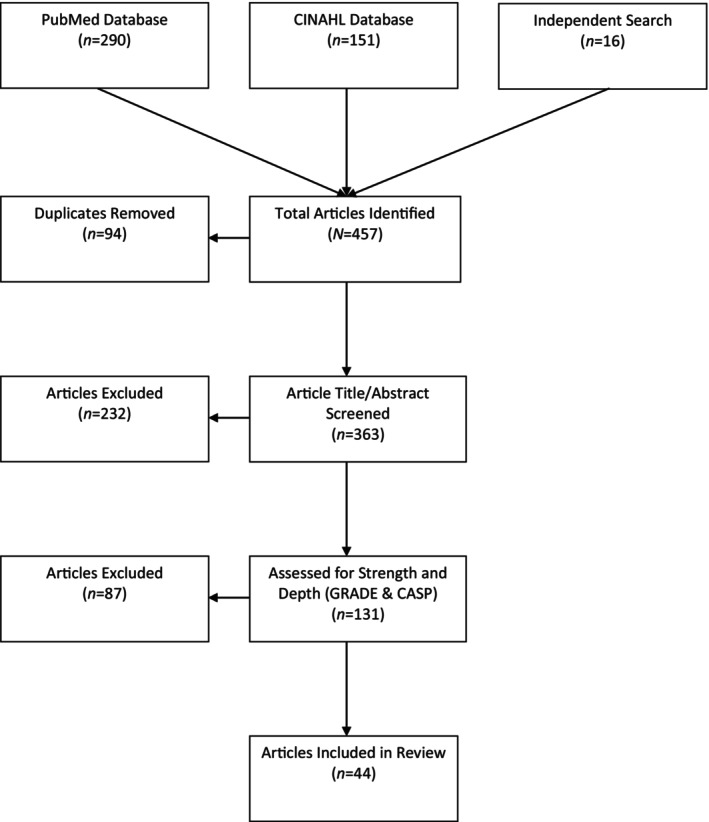
Systematic literature review process.

A literature review was conducted using PubMed and CINAHL databases. Key terminologies related to homelessness and resiliency were used without date restrictions, resulting in 441 articles (151 from CINAHL and 290 from PubMed). Cross‐referencing added 16 more studies, totalling 457 articles. Inclusion criteria required English articles focusing on conceptual or theoretical frameworks for resiliency, interventional studies within homelessness, qualitative analyses and peer‐reviewed publications. Exclusion criteria removed duplicates (94 articles) and irrelevant titles/abstracts (232 articles), leaving 131 articles. Further review excluded 87 articles for peripheral relevance or low quality. The remaining 44 articles were assessed using the GRADE approach and CASP checklists to evaluate evidence quality and methodological integrity. These 44 articles formed the foundation for the in‐depth analysis of resiliency in homelessness. Figure 1 provides a summary of the evidence search. Additionally, Appendix S1 provides a detailed overview of the analysis.

## 3 Conceptual Analysis

The extant literature supports that resiliency is a complex construct extensively examined across many scholarly fields. It is significantly relevant to the adversities encountered by individuals experiencing homelessness. The global prevalence of homelessness necessitates a nuanced comprehension of the adaptive strategies and strengths affected individuals leverage.

Discussions on resiliency are prevalent in allied health across nursing, social sciences, medicine and public health. However, the specific exploration of resiliency as it pertains to individuals in a state of homelessness remains relatively underrepresented. This analysis endeavours to further elucidate the complex dimensions of resiliency within the homeless context, with a pronounced focus on the nursing discipline. Table 2 presents a summarised alignment of the literature within Roger's framework.

**TABLE 2:** Literature alignment with Roger's evolutionary framework.

Roger's conceptual category	Key references	Description
Surrogate terms	Llistosella et al. (2022); Foroughi et al. (2022); Gartland et al. (2019); Mao et al. (2022); Martins (2008)	Resiliency in homelessness is a multifaceted concept, encompassing terms like ‘coping capacity’, ‘adaptive strength’ and ‘resourcefulness’. These reflect diverse strategies for managing stress and adversity, transcending mere survival to embody psychological balance and resource optimisation, crucial for holistic nursing understanding and support
	Heathcote, Wullschleger, and Sun (2019); Mabhala, Yohannes, and Griffith (2017); Larkin, Beckos, and Shields (2012)	
Antecedents	Karadzhov, Yuan, and Bond (2020); Nevard et al. (2021); Mao et al. (2022); Slockers et al. (2018); Aidala et al. (2016); Larkin, Beckos, and Shields (2012); Paul et al. (2018)	Antecedents of resiliency in homelessness include individual psychological foundations like self‐esteem, emotional intelligence, coping mechanisms, physical health, social networks, economic stability, access to resources and education. These elements represent the underlying conditions that either contribute to or hinder the development of resilience in individuals facing homelessness
Attributes	Zhang, Wu, and Slesnick (2021); Finfgeld‐Connett (2010); Kirst et al. (2014); Mejia‐Lancheros et al. (2021); Karadzhov, Yuan, and Bond (2020)	Resiliency attributes in the homeless context include adaptability, problem‐solving abilities, hope and social support. These qualities and capabilities enable individuals to endure and navigate the challenges of homelessness. They encompass the emotional, cognitive and social strengths that individuals draw upon in their daily lives
Consequences	Cush et al. (2020); Mitchell et al. (2023); Nevard et al. (2021); Ellis, Dietz, and Chen (2022); Liu et al. (2020)	Consequences of resilience among the homeless include elevated self‐awareness, emotional exhaustion, interpersonal bonding, dependency on specific resources or environments and adaptations to urban landscapes. These are the outcomes of resilience, both positive and negative, impacting the individual's life and their interaction with the community and environment

*Note:* Rodgers and Knafl (2000).

Before delving into the intricacies of resiliency within the context of homelessness, it is imperative to undertake a critical review of associated terminologies and surrogate concepts to ensure a thorough understanding of the concept's breadth and scope. This foundational review will set the stage for a more informed and comprehensive analysis of resiliency in the context of homelessness, enhancing the theoretical and practical approaches to supporting this vulnerable population.

### 3.1 Surrogate Terms for Resiliency

Resiliency is an intricate concept characterised by many surrogate or analogous terms, each reflecting a facet of the concept but not fully encapsulating its complexity. The construct is evident across diverse dimensions of human and societal interaction (Llistosella et al. 2022; Foroughi et al. 2022; Gartland et al. 2019). Detailed scrutiny of resilience's proxy terms uncovers a range of linguistic subtleties across the physical, psychological and existential domains, meriting attention.

Within allied health literature, a detailed lexicon surfaces upon examination of the nuanced dynamics of resilience among populations experiencing homelessness. Terms such as coping capacity and adaptive strength emerge, articulating the essence of an individual's proficiency in managing stress and fluctuating life conditions (Mao et al. 2022). Nursing literature acknowledges the intrinsic vigour that drives many to persist through formidable challenges (Martins 2008). Nevertheless, resilience encompasses more than persistence; it involves sustaining psychological balance amid the upheavals of homelessness while navigating towards health‐promoting behaviours (Heathcote, Wullschleger, and Sun 2019).

Furthermore, empirical studies on the lived experiences of homeless individuals reveal pronounced resourcefulness, evidencing their ingenuity in optimising scarce resources (Mabhala, Yohannes, and Griffith 2017). The concept of recovery ability, or the potential to recover from adversity, is also pronounced, particularly in the face of significant obstacles (Larkin, Beckos, and Shields 2012). Collectively, these terms converge to form a broader construct of adaptive responsiveness that transcends basic survival, offering a complex perspective through which nursing professionals can more deeply comprehend, empathise with and effectively assist individuals facing homelessness. The current discourse necessitates a further investigation into the specific attributes of resilience.

Engaging with resilience via an evolutionary method of concept analysis reveals a constellation of surrogate terms that enhance our comprehension of this layered construct. Such terms, including coping capacity, adaptive strength, resourcefulness and recovery ability, are not simply interchangeable; they represent the diverse experiential and observational dimensions of resilience among those experiencing homelessness. These terms encompass the array of survival and flourishing strategies individuals employ in challenging environments. Acknowledging these surrogates within the nursing context facilitates a holistic assessment of resilience concerning homelessness, offering insights that extend beyond conventional paradigms and into a profound acknowledgement of human capacity for endurance and adaptation.

### 3.2 Attributes of Resiliency

Resiliency takes on a poignant depth when viewed through the lens of homelessness. Several attributes of resiliency enable individuals to persistently navigate and transcend the challenges of being without a permanent and stable residence. The foremost attributes of resiliency in the context of homelessness described in the literature include adaptability, coping, problem‐solving ability, social support and hope. All these attributes present a framework for the concept of resiliency that opposes the challenges faced by people experiencing homelessness.

#### 3.2.1 Adaptability

Adaptability emerges as a pivotal attribute for individuals experiencing homelessness. The lack of a permanent shelter necessitates an enhanced capacity for adaptation to continuously evolving situations (Noh and Choi 2020; Bradley, McGowan, and Michelson 2018). Individuals in these circumstances must frequently modify their daily routines, thought processes and behaviours to cope with the inherent uncertainties of their situation. This adaptability is closely interwoven with emotional resilience, particularly in maintaining emotional stability amid the challenging environment of homelessness. Effective emotion regulation strategies are critical, especially for mitigating the chronic stress and societal stigma associated with homelessness (Karadzhov, Yuan, and Bond 2020; Gartland et al. 2019). Additionally, the capacity to manage stress, emotional discernment and achieve psychological peace is crucial, with emotional dysregulation often contributing significantly to the perpetuation of homelessness (Mitchell et al. 2023).

#### 3.2.2 Problem‐Solving

Additionally, problem‐solving abilities are also vital. Homeless individuals must use advanced problem‐solving methods to deal with daily challenges, such as finding safe sleeping locations and accessing resources (Zhang, Wu, and Slesnick 2021). Critical thinking is imperative in discerning, accessing and leveraging available resources, which can significantly enhance an individual's inherent coping capabilities (Finfgeld‐Connett 2010).

#### 3.2.3 Hope

Hope is an indispensable component of resilience among those facing homelessness. Despite severe adversity, these individuals often exhibit an extraordinary ability to endure stress without losing hope (Finfgeld‐Connett 2010). Self‐reflection is crucial in this context, enabling individuals to consider their life trajectories, assess their current situation and envision a stable future. This reflective capacity fosters hope, a dynamic force in bolstering resilience, both before and after engagement with housing initiatives (Kirst et al. 2014).

#### 3.2.4 Societal Networks

The nature and strength of social supports available in the community play a crucial role in fostering resilience among people experiencing homelessness. Unfortunately, traditional social ties, such as family and friends, may be limited or non‐existent, especially among homeless youths (Embleton et al. 2016). In such cases, the immediate social network may comprise other homeless individuals, support groups and service outreach workers. The quality of these social supports and the broader community's attitude towards homelessness can significantly influence the individuals' ability to cope with and overcome the challenges associated with homelessness. Positive, compassionate and inclusive community attitudes and robust support systems can enhance homeless individuals' sense of belonging and self‐worth, promoting their mental and emotional well‐being. Conversely, negative perceptions and inadequate support can exacerbate feelings of isolation and hopelessness, hindering their efforts to achieve stability and reintegration into society.

### 3.3 Antecedents of Resiliency

The concept of resiliency, especially when viewed through the lens of homelessness, unfolds as an intricate tapestry woven from various threads. For those facing the unforgiving realities of homelessness, resiliency is not just a desirable trait but often a matter of survival. Grasping the antecedent factors that underpin resiliency offers a nuanced understanding of the experiences and needs of this vulnerable population. When one investigates these antecedents deeper, one quickly realises the central role of individual attributes.

#### 3.3.1 Innate Coping Ability

At the heart of resiliency lies the individual's psychological foundation. This foundation comprises self‐esteem, emotional intelligence and a sense of purpose, which often determines the ability to navigate daily adversities in homelessness. Coping mechanisms, honed over time and experience, become the linchpins of survival, aiding decision‐making, stress management and response to threats (Karadzhov, Yuan, and Bond 2020; Paul et al. 2018). Physical health remains a crucial factor while continuously under siege due to exposure and limited access to healthcare. Maintaining health amid such challenges is a testament to the incredible adaptability and perseverance of those who are homeless (Slockers et al. 2018). Equally remarkable is the capacity to seek and cultivate relationships even in the harshest circumstances.

#### 3.3.2 Presence of Individual Social Networks

While homelessness often leads to societal isolation, social connections within this community can serve as critical support pillars (Nevard et al. 2021). As traditional family bonds may diminish or evolve, individuals experiencing homelessness form new, meaningful relationships with others sharing similar circumstances. These connections, rooted in shared struggles and mutual empathy, provide essential emotional support and practical advice. Additionally, a sense of belonging fostered within shelters, support groups or outreach programmes offers a vital counterbalance to the broader societal exclusion, reinforcing self‐worth and providing much‐needed moments of comfort.

The dynamics of resilience in the context of homelessness indicate that social isolation does not equate to a complete disconnection from social ties. Within the homeless community, unique and supportive social networks often emerge (Tsai and Rosenheck 2015; Heaslip et al. 2021a, 2021b). While different from traditional family structures, these networks are no less significant. They may even form complex, supplementary social frameworks offering critical support (Heaslip et al. 2021a, 2021b). The shared experience of homelessness fosters deep connections as individuals relate to each other's struggles, creating bonds that are crucial for emotional resilience.

Moreover, community belonging is instrumental in cultivating resilience among people experiencing homelessness. This sense of belonging can arise in various environments, such as shelters, support groups or outreach initiatives (Marshall et al. 2020). These places provide a sense of inclusion and community that is often lacking in the wider society. They serve as vital reminders of individual value, contributing to psychological well‐being by offering opportunities for respite and reflection, which are essential for maintaining mental health and fostering resilience.

#### 3.3.3 Environmental Factors

The environments and external conditions that homeless individuals encounter daily are equally influential. The physical spaces where they find shelter, the availability of resources and the societal attitudes they confront can hinder or promote resilience. Hostile environments can exacerbate feelings of exclusion, while supportive settings can facilitate coping mechanisms and offer pathways to recovery and stability (Aubry et al. 2020).

The environmental factors influencing resiliency among people without housing are immediate and systemic. Economic stability, while elusive, is a beacon many strive towards, with financial challenges often being the initial catalyst propelling them into homelessness. While primarily focused on unhoused individuals with HIV, Aidala et al. (2016) reinforce this claim in a systematic review of the economic influences on homelessness. Their article supports the claim that access to financial resources contributed to overall resiliency. Additionally, access to education might be sporadically available. However, it remains a potent tool for change and may lead to increased resiliency. In their systematic review of protective factors in homelessness, Grattan et al. (2022) report that education and a supportive family were protective factors in youth homelessness resiliency. These resources address immediate needs and illuminate the broader socio‐environmental context that shapes resiliency.

Understanding the antecedents of resiliency within the realm of homelessness unravels this community's profound strength, adaptability and tenacity. However, these antecedents are not just the building blocks of resiliency but are intertwined with the consequences that resiliency yields. As we discuss these consequences, it becomes vital to remember that resiliency, while a protective shield, also has its toll. The ramifications of sustained resiliency amid continuous adversity lay the groundwork for a deeper exploration of the lived experiences of those facing homelessness.

### 3.4 Consequences of Resiliency

The concept of resiliency, particularly concerning homelessness, does not merely end with understanding its antecedents. Instead, it propels us into the realm of consequences, both positive and challenging, that emerge as byproducts of this relentless resiliency.

#### 3.4.1 Elevated Self‐Awareness

Resiliency cultivated amid the adversities of homelessness often results in an elevated sense of health and self‐awareness, as evident in the work of Irish researchers Cush et al. (2020). This heightened cognizance of one's strengths and vulnerabilities can shape personal narratives, making individuals more adept at handling challenges. This self‐awareness is evident and influenced from an early age. Liu et al. (2020), in a study of resiliency in 565 adults experiencing homelessness, concluded that resiliency is a positive factor in overcoming adverse childhood experiences (ACEs). In other words, the resiliency had a mitigating effect on the consequences of ACEs in this population (Liu et al. 2020).

#### 3.4.2 Emotional Exhaustion

However, this continuous resiliency can also lead to emotional and physical exhaustion. The constant need to adapt, confront and overcome might leave little room for emotional recovery. Physically, too, the toll can be palpable. While initial resiliency may lead to innovative solutions for survival, over time, the wear and tear on the body due to recurrent stressors can have detrimental health effects. This physical impact, however, is just one facet, as the ripple effects of resiliency stretch beyond the individual to the larger community (Durbin et al. 2019; Mitchell et al. 2023).

#### 3.4.3 Interpersonal Bonding

Within the social sphere, resiliency demonstrated by those experiencing homelessness can foster profound relationships (Nevard et al. 2021). Bonds cemented through shared hardships often possess a depth of understanding and empathy unparalleled by more traditional relationships. These networks can offer a unique sense of community, a shared identity rooted in a collective experience.

Conversely, consistent resiliency and extreme self‐sufficiency can sometimes lead to social isolation. The constant fight for survival might make some wary of new relationships or distance themselves from others to shield their vulnerabilities (Durbin et al. 2019). Following the challenges and opportunities within social connections, it is essential to consider the direct impact of resiliency on the immediate environment of those experiencing homelessness.

#### 3.4.4 Dependency

Environmentally, the resilient strategies adopted by those facing homelessness can lead to creating or identifying safer havens within urban landscapes, such as preferred spots to rest or communities that offer only a minimum of safety (Ellis, Dietz, and Chen (2022). This adaptation to the environment might influence urban planning or community outreach strategies. However, continuous resiliency might also lead to an increased dependency on specific resources or shelters, potentially hindering the exploration of more sustainable solutions. Moreover, the inherent dynamism between individuals and their environment showcases the intricate balance of adaptability and dependency (Hudson et al. 2016). As resilient strategies evolve to match the ever‐changing urban landscapes, they also reshape the fabric of these environments and the societal perceptions surrounding homelessness.

### 3.5 A Model Case

One component of Roger's evolutional method is to create a model case to bring the elements of the concept under investigation to the forefront (Rodgers and Knafl 2000). In the following case, the attributes and antecedents of resiliency will be described, hopefully elucidating the intertwined consequences. As the model case is presented, it also helps to emphasise the value of such illustrative narratives in bringing abstract concepts to life, offering a humanistic lens through which we can comprehend the intricate nature of resiliency within the realm of homelessness. This model can either be a real‐world example or a hypothetical creation. This model case is wholly fabricated for this analysis. Still, it highlights the possible realities of a person's experience with homelessness and resiliency.

#### 3.5.1 Case Study

Jane, a 45‐year‐old woman, has navigated the tumultuous journey of homelessness for 3 years after losing her job and being evicted. Facing harsh weather, food scarcity and safety issues, Jane has also grappled with societal stigma, leading to feelings of isolation and dehumanisation (Bradley, McGowan, and Michelson 2018). Despite these hurdles, Jane's resilience shines through.

Her resilience manifests in diverse coping strategies and adaptive behaviours. Jane's coping capacity is highlighted in her ability to find shelter in adverse weather, a testament to her resourcefulness (Mao et al. 2022). Her adaptive strength is visible in her innovative use of materials like newspapers for insulation and creating makeshift shelters, reflecting her ability to improvise effectively in challenging situations (Karadzhov, Yuan, and Bond 2020). Moreover, Jane's recovery ability is evident in how she overcomes food scarcity, displaying ingenuity in securing food (Mabhala, Yohannes, and Griffith 2017).

Jane's story is a powerful example of the resilience individuals experiencing homelessness can exhibit. It embodies the capacity to adapt and survive amid significant adversities.

##### 3.5.1.1 Manifestations of Resilience Attributes

Jane's adaptability, a crucial aspect of her resilience, is exemplified by her constant adjustments to routines in response to available resources and safety needs, akin to the adaptive behaviours noted in Mao et al. (2022). Her emotional stability, particularly evident in her composed response to confrontations, reflects the emotional resilience and awareness discussed in Heathcote, Wullschleger, and Sun (2019). Jane's enhanced problem‐solving skills, crucial for finding safe rest areas and accessing food, resonate with findings by Mabhala, Yohannes, and Griffith (2017), highlighting the resourcefulness of individuals experiencing homelessness.

Her transformed social support network, now inclusive of connections within the homeless community, aligns with insights from Larkin, Beckos, and Shields (2012), emphasising the significance of collaborative resource sharing and mutual protection in fostering resilience. Jane's persistent hope and continuous search for betterment through employment or community resources echo the resilience traits discussed in Martins (2008), demonstrating an unwavering determination to improve her circumstances despite the adversities of homelessness.

##### 3.5.1.2 Antecedents and Resilience Development

As reflected in her journey through homelessness, Jane's resilience is deeply rooted in personal qualities like self‐esteem and emotional intelligence, which are critical components for effectively managing the challenges of being unhoused (Martins 2008). Her adaptability is a pivotal strength despite the toll on her physical health. The skills she developed in her previous job roles sporadically helped her earn income, highlighting her resourcefulness and ability to leverage past experiences in adverse situations (Mao et al. 2022).

Additionally, Jane's integration into the community of those experiencing homelessness has fostered new connections. These relationships offer mutual support and a sense of belonging, which is crucial for maintaining psychological well‐being amid the hardships of homelessness (Heathcote, Wullschleger, and Sun 2019; Mabhala, Yohannes, and Griffith 2017). Her story exemplifies how resilience in the face of homelessness is not just about individual traits but also about the strength of community bonds and the ability to adapt to new circumstances.

##### 3.5.1.3 Consequences of Sustained Resilience

Jane's journey of resilience, marked by heightened self‐awareness and skill in navigating daily challenges, echoes the intricate nature of resilience in homelessness, as outlined by researchers like Heathcote, Wullschleger, and Sun (2019). However, her continual resilience also brings about emotional exhaustion, a phenomenon recognised in the works of Durbin et al. (2019). Through her experiences, Jane develops profound connections with others in similar situations, fostering a unique sense of community, a concept explored by Mabhala, Yohannes, and Griffith (2017).

The adaptations Jane makes to her environment and routines, including resting in public spaces and altering her safety practices, reflect the practical aspects of resilience highlighted by Mao et al. (2022). Nonetheless, her reliance on certain routines may limit her potential for finding more sustainable solutions, a concern raised in the literature by Martins (2008).

Jane's story illustrates the multifaceted resilience that those experiencing homelessness exhibit, marked by adaptability, emotional stability, problem‐solving skills, social support and hope. These qualities underscore the complexities of life without stable housing, as discussed by Larkin, Beckos, and Shields (2012). Her experience highlights the critical need for a deep understanding and support for homeless individuals, shedding light on the layered dimensions of resilience in such challenging circumstances.

## 4 Discussion

The resiliency journey within the homelessness context, marked by its complexities and challenges, showcases the indomitable human spirit and underscores the need for comprehensive support systems, interventions and societal understanding. As a survival mechanism and testament to human tenacity, this aspect of resiliency is especially pertinent in nursing, where empathy and understanding are crucial in addressing the needs of those unhoused (Mao et al. 2022; Martins 2008).

### 4.1 Special Considerations for Those Experiencing Homelessness

The unique nature of the homeless under investigation in the reviewed literature reveals a broad spectrum of reasons for homelessness, including mental health issues, substance use disorders, economic hardships and social disruptions. These factors contribute to the instability of housing situations and influence the manifestation of resilience among those individuals.

#### 4.1.1 Impact of Mental Health and Substance Use Disorders

Mental health issues and substance use disorders significantly affect the ability of those experiencing homelessness to form and maintain social connections, a critical component of resilience. Individuals struggling with these issues often face compounded challenges, including stigmatisation, reduced access to services and difficulties in sustaining employment and relationships. These factors can undermine traditional forms of resilience, such as adaptability and problem‐solving skills, by impairing cognitive and emotional functioning (Karadzhov, Yuan, and Bond 2020; Durbin et al. 2019).

Despite these challenges, resilience attributes do emerge among this population, albeit in different forms. For instance, peer support networks within the homeless community often serve as critical sources of social support, providing emotional and practical assistance that can enhance resilience (Kennedy et al. 2022). These networks help mitigate the isolation caused by mental health and substance use issues, fostering a sense of belonging and mutual aid.

#### 4.1.2 Variations Across Subpopulations

Different subpopulations within the homeless community exhibit varying resilience attributes based on their specific circumstances. For example, homeless youth often display remarkable adaptability and resourcefulness in navigating their environments. However, their lack of traditional social support, such as family, can pose significant challenges (Embleton et al. 2016). Older adults experiencing homelessness may rely more on established social networks and community services to bolster their resilience (Cush et al. 2020).

The resilience attributes identified in the literature review, such as adaptability, problem‐solving and hope, appear to be universally present among homeless individuals regardless of specific factors like mental health or substance use issues. However, the expression and effectiveness of these attributes can vary. For instance, mental health issues may require more robust external support to enable individuals to leverage their inherent resilience effectively (Mitchell et al. 2023).

#### 4.1.3 Influence of Environmental and Societal Factors

The broader community's attitude towards homelessness and the availability of supportive services are pivotal in shaping resilience among those experiencing homelessness. Positive societal attitudes and inclusive policies can enhance resilience by providing necessary resources and reducing stigmatisation. Conversely, negative perceptions and inadequate support exacerbate the difficulties faced by homeless individuals, potentially diminishing their resilience (Aubry et al. 2020).

While the core resilience attributes such as adaptability, problem‐solving and hope are consistent across homeless populations, their manifestation is influenced by factors such as mental health issues, substance use disorders and the broader societal context. Recognising these variations is crucial for developing tailored interventions that address the unique needs of different subpopulations within the homeless community, ultimately fostering a more supportive and resilient environment for all individuals experiencing homelessness.

### 4.2 Philosophical and Societal Dimensions of Resilience in Homelessness

Philosophically, resiliency in homelessness invites deep contemplation on the essence of humanity and societal structures, posing questions about its nature—whether it is an innate human trait or shaped by external challenges and societal perceptions. The findings suggest that resiliency combines inherent qualities and learned responses, a blend of innate resilience and adaptability shaped by personal strength and societal influences (Llistosella et al. 2022; Foroughi et al. 2022).

In societies that often equate worth with material success, the resilience displayed by individuals experiencing homelessness calls for re‐evaluating these standards. Through the lenses of both science and philosophy, the concept of resilience in homelessness reveals a rich spectrum of insights. This dual approach—scientific inquiry and philosophical reflection—provides a comprehensive and nuanced understanding of resilience, highlighting its tangible and intangible aspects (Gartland et al. 2019; Heathcote, Wullschleger, and Sun 2019).

This analytical approach emphasises the importance of recognising the multifaceted nature of resilience in homelessness and its implications for societal attitudes, policymaking and healthcare practices, particularly in nursing. It advocates for a holistic understanding that respects and nurtures the resilience of individuals facing one of society's most pressing challenges.

### 4.3 Implications for Nursing

Exploring resilience in nursing concerning homelessness is pivotal, casting a new light on patient care, research, education and advocacy. As this concept takes centre stage, it necessitates a manifold approach, intertwining the expertise of various disciplines to deepen our understanding and craft interventions that are as robust as they are nuanced. From the granular level of individual care to the broader strokes of policy advocacy, resilience invites a re‐examination of traditional practices. It is a call to integrate psychological well‐being into the nursing fabric, empowering practitioners to support those facing the adversities of homelessness with reactive and proactive strategies.

#### 4.3.1 Implications for Nursing Research

In nursing research, exploring resiliency opens pathways for future studies, laying a solid foundation for nurse‐led research. The complexity of resiliency, characterised by the interaction of internal strengths and external influences, necessitates a collaborative research approach. This interdisciplinary challenge calls for nursing researchers to partner with psychology, sociology and public health experts, thereby expanding the breadth and depth of the investigation (Martins 2008; Mao et al. 2022).

The multifaceted nature of resiliency invites a methodological evolution, integrating qualitative insights with quantitative rigour, effectively capturing the full scope of the phenomenon (Heathcote, Wullschleger, and Sun 2019). Such a deep understanding of resiliency also lays the groundwork for developing or refining assessment tools designed to accurately reflect the subtleties of resilience in various groups, particularly those experiencing homelessness (Mabhala, Yohannes, and Griffith 2017; Larkin, Beckos, and Shields 2012). This approach enhances the understanding of resilience and contributes significantly to developing targeted interventions and supportive strategies for the homeless population.

#### 4.3.2 Implications for Nursing Practice

In healthcare and nursing practice, the insights gained about resiliency enable a more comprehensive approach to patient care. Understanding the depth of resiliency empowers nurses to consider the immediate physical needs and the psychological aspects of patient care. Chan et al. (2018) illustrate this through a community‐based intervention that showcases the complexities of caring for homeless individuals and the effectiveness of interprofessional care. Their approach mirrors the detailed inpatient care in an outpatient setting, yielding positive results (Chan et al. 2018). This holistic view aids in creating both reactive and proactive interventions to foster resilience in those they assist.

Acknowledging the diverse factors influencing resilience enables personalised care, addressing the person's unique history, challenges and strengths. Nurses with this knowledge can also lead educational initiatives to cultivate resilience within communities, particularly among vulnerable groups. Furthermore, this understanding of resilience extends to advocacy roles, where nurses can use their insights to influence policies that accurately reflect the needs and potential of the individuals they care for, ensuring a more empathetic and effective healthcare system.

#### 4.3.3 Implications for Nursing Education

The intricate understanding of resiliency, particularly in the context of homelessness, holds significant implications for nursing education. Educators must integrate the concept of resiliency into nursing pedagogy, creating a curriculum that addresses its complexities and equips students to foster resilience in diverse populations (Mao et al. 2022). This approach prepares nursing students to recognise and enhance resilience among vulnerable groups, including people experiencing homelessness.

Nursing education must also prioritise interprofessional collaboration, reflecting the interdisciplinary nature of resiliency research (Larkin, Beckos, and Shields 2012). This approach prepares students for effective teamwork in healthcare settings. Educational programmes should include training in qualitative and quantitative research methods, enabling future nurses to contribute to and utilise ongoing research on resiliency and homelessness (Mabhala, Yohannes, and Griffith 2017).

Also, incorporating case studies and simulations on homelessness and resilience into coursework is essential. This provides students with realistic scenarios emphasising empathy in care (Heathcote, Wullschleger, and Sun 2019). This inclusion helps students understand the practical aspects of resilience in real‐life contexts.

Finally, developing assessment competencies is crucial, allowing nursing students to identify levels of resilience and the factors influencing it across populations. Simulation‐based learning experiences effectively develop these skills in a controlled environment before students apply them in clinical settings (Martins 2008). Such educational strategies prepare nursing students to be clinically adept and empathetic, understanding the profound impact of their role in fostering individual and community resilience, especially among the homeless and underserved.

#### 4.3.4 Implication for Nurse Advocacy

Lastly, the expanded understanding of resiliency should be channelled into leadership and policy formulation, enabling nurses to champion systemic changes that bolster the health and well‐being of marginalised groups, including people without housing. This comprehensive approach to nursing advocacy is pivotal for evolving a healthcare system that tackles the physical, psychological and societal aspects impacting patient health and recovery (Mabhala, Yohannes, and Griffith 2017; Heathcote, Wullschleger, and Sun 2019).

Understanding resilience allows nurses to reflect on their professional journey, enhancing self‐awareness and reinforcing their dedication to their role. Integrating research, practice, education, advocacy and resilience in nursing serves as both a guide and an impetus. It influences patient care, shapes interventions, stimulates research and reinvigorates the commitment to comprehensive, empathetic and informed nursing care, which is particularly crucial for those confronting the hardships of homelessness (Larkin, Beckos, and Shields 2012; Martins 2008).

This perspective encourages nurses to employ resilience as a concept in care and a foundational element in their professional ethos. They are empowered to advocate for and implement policies that reflect a deep understanding of the complexities faced by homeless populations. Such an approach underscores the significant role of nurses as crucial agents in promoting health equity and resilience among society's most vulnerable.

Overall, the intricate understanding of resiliency, especially concerning the vulnerable population of people without housing, has significant implications for nursing education. As educators shape the curricula, they must integrate the concept of resiliency into the framework of nursing pedagogy. This integration demands a curriculum that addresses the complexities of resiliency, ensuring nursing students acquire the knowledge and skills to recognise and foster resiliency in diverse populations.

### 4.4 Limitations

This concept analysis has several limitations. Firstly, it relies on existing literature, potentially introducing selection bias and overlooking non‐English research. The focus on Rogers's evolutionary method may limit the exploration of other theoretical frameworks. Additionally, the analysis centres primarily on adults, possibly missing nuances in resiliency among youth, families or the elderly. While highlighting individual strengths, it may underemphasise structural factors contributing to homelessness. Future research should include a broader range of studies, different theoretical perspectives and diverse subpopulations. Longitudinal and interventional studies are needed to understand resiliency development and the effectiveness of specific programmes and policies, with cross‐disciplinary collaborations essential for comprehensive support systems.

## 5 Conclusion

This concept analysis offers a nuanced understanding of resiliency in homelessness, using Rogers's evolutionary method to highlight the interplay among individual, social and environmental factors. Resiliency is a complex, adaptive process, involving emotional stability, problem‐solving skills, social support and hope, which help homeless individuals navigate significant challenges. The concept of resiliency has evolved from material science to major roles in psychology and public health, framing it as a positive adaptation mechanism shaped by individual strengths and environmental factors. This understanding emphasises the need for compassionate, strength‐based care in nursing. It suggests targeted interventions to support homeless individuals' resilience.

However, there are still gaps in the literature. Future research should focus on longitudinal studies to understand how resiliency develops over time and on interventional studies to explore the effectiveness of specific programmes and policies. Cross‐disciplinary collaborations are essential to deepen our understanding and develop comprehensive support systems for homeless individuals.

By expanding the knowledge base on resiliency in homelessness, this analysis contributes to academic discourse. It informs practical applications in healthcare, particularly nursing. It advocates for a holistic approach to care that recognises and nurtures the resilience of individuals facing homelessness, ultimately promoting their well‐being and dignity.

## Author Contributions

All the authors have made substantial contributions to conception and design, acquisition of data or analysis and interpretation of data; drafted the manuscript or involved in critical revision for important intellectual content; approved the final version to be published; participated sufficiently in the work to take public responsibility for appropriate portions of the content; agreed to be accountable for all aspects of the work in ensuring that questions related to the accuracy or integrity of any part of the work are appropriately investigated and resolved.

## Conflicts of Interest

The authors declare no conflicts of interest..

### Peer Review

The peer review history for this article is available at https://www.webofscience.com/api/gateway/wos/peer‐review/10.1111/jan.16440.

## Supporting information


Appendix S1.

## Data Availability

The data that supports the findings of this study are available in the supplementary material of this article.
