# Supplementary material for: Resiliency in Persons Experiencing Homelessness: A Concept Analysis Using the Evolutionary Framework
Source: J Adv Nurs. 2024 Sep 10;81(2):749–61. doi: 10.1111/jan.16440 (PMC11730778; doi:10.1111/jan.16440)
Supplement: Supplementary file 1 — Appendix S1. [file JAN-81-749-s001.docx]

**Appendix A: Literature review summary for the concept of resiliency within the context of homelessness using GRADE and CASP tools.**

| **Author** | **Year** | **Primary Insights/Notes** | **Type of Evidence** | **Evidence Quality/Strength** |
| --- | --- | --- | --- | --- |
| Aidala et al. | 2016 | Housing as a Health Determinant: Housing is identified as a key factor affecting health outcomes in people with HIV, linking larger socio-economic conditions to individual health.  Association with Health Outcomes: Research indicates that poor housing status correlates with poorer health outcomes, medication adherence, and increased risk behaviors among HIV-positive individuals.  Evidence from Studies: Data from 152 studies show a consistent pattern where inadequate housing is independently linked to negative health outcomes, despite various patient and healthcare system factors.  Importance of Intervention: The evidence suggests that interventions to improve housing stability for people with HIV can significantly enhance health outcomes and treatment efficacy. | Systematic Review | High/Strong |
| Aubry et al. | 2020 | Housing Stability Increased: Permanent supportive housing significantly increased long-term housing stability for homeless individuals with moderate to high support needs in high-income countries, demonstrating its effectiveness over usual care.  Mixed Results on Health and Employment: While permanent supportive housing did not show measurable effects on psychiatric symptoms, substance use, income, or employment, income interventions that combined housing subsidies with case management resulted in more days stably housed. The impact on mental health and employment, however, was not clear.  Need for Long-Term Research: There's a call for more comprehensive research to understand the long-term effects of housing and income interventions on health, substance use, and quality of life, indicating that current knowledge on these aspects is not conclusive.  Economic Analysis Required: The review included studies on cost and cost-effectiveness but did not provide a clear synthesis in this summary, suggesting a need for detailed economic analyses to inform policy and funding decisions related to these interventions. | Systematic Review | High/Strong |
| Bradley et al. | 2018 | Impact on Parental Self-concept and Mental Health: Homelessness can significantly affect parents’ perceptions of themselves in their role as caregivers, often leading to a negative self-concept. This challenge is further exacerbated by issues related to mental health, as the stresses of homelessness can induce or aggravate psychological distress, which in turn can impact the quality of parenting.  Challenges to Resources and Autonomy: The scarcity of material resources and the environmental constraints of homelessness place considerable pressure on parents, undermining their autonomy and self-efficacy. These conditions make it difficult for parents to provide for their children's needs and maintain their authority, which are vital for effective caregiving.  Adaptive Parenting Strategies: Despite these adversities, homeless parents often employ adaptive strategies to mitigate the negative effects of their situation. These include maintaining a positive outlook, valuing their role as parents, and devising practical solutions to navigate the challenges of parenting while homeless. This resilience is crucial for sustaining the parent-child relationship and ensuring the well-being of children in such precarious situations. | Systematic Review | High/Moderate |
| Chan et al. | 2018 | Necessity of Targeted Interventions: There's a recognized need for specific interventions that cater to PLWH who face HUH. This is crucial for reducing new HIV infections and improving individual health outcomes.  Lack of Comprehensive Literature: Despite the necessity, there's an absence of a compiled body of literature that specifically addresses the unique challenges faced by PLWH with HUH. This gap indicates that more research and compilation of existing studies are needed to inform effective clinic-based programs.  Mixed Results from Current Interventions: The review suggests that singular interventions, such as case management, patient navigation, financial incentives, and mobile technology, have shown mixed results. They often fail to address the multifaceted barriers to care that this vulnerable population encounters.  Need for Multi-Component Interventions: There is an emphasis on the need for multi-component interventions to adequately address the complex needs of PLWH with HUH. However, there's an acknowledged gap in knowledge and data, which may hinder the widespread adoption of such interventions.  Future Research Directions: The review highlights the importance of including implementation outcomes in future research. This is to ensure that interventions for PLWH experiencing HUH can be effectively adapted across various clinical settings, potentially leading to better health outcomes for this population. | Quasi-experimental | Moderate |
| Crittenden | 1985 | Infant Congenital Characteristics: Maltreated infants do not show differences in congenital characteristics when compared to non-maltreated control infants, suggesting that maltreatment is not related to inherent differences in the children’s nature or genetic make-up.  Learned Behavior Patterns: The deviant behavior exhibited by maltreated infants appears to be learned rather than congenital. This implies that the maltreatment and its environment influence the infants' behavior, rather than the behavior being a result of innate characteristics.  Resilience and Intervention: The study found that after intervention directed at the mothers, the infants demonstrated behavioral improvement. This suggests that maltreated infants are resilient and capable of positive change when their environment is improved, particularly when the maltreatment behaviors of the mother are addressed. | Quasi-experimental | Moderate |
| Cush et al. | 2020 | Marginalization and Health Disparities: The position of marginalized groups like older Irish Travellers and older homeless individuals is often overlooked in positive health and aging agendas. There is a need to recognize and address the unique challenges these populations face, which can range from social exclusion to access to health care, to ensure equitable later-life experiences​​.  Heterogeneous Health Trajectories: There is an indication of increasingly heterogeneous health trajectories within these groups, suggesting a wide variance in health outcomes and experiences among individuals. This diversity within the groups underscores the complexity of their needs and the necessity for personalized approaches to health and social care​​.  Scoping Review Findings: A scoping review has been conducted to synthesize international research on the life-course and structural determinants of positive subjective health for these populations. This review spans publications from 1998 to 2020 and has repeated iterations to capture the most current state-of-the-art knowledge. It included 38 publications in the final sample, which provided evidence of specific life-course and structural factors that are influential for both groups | Systematic Review | High/Moderate |
| Durbin et al. | 2019 | Resilience and Stress Over Time: The study found that over the 24-month period, resilience levels of the participants increased while their perceived stress levels decreased. This suggests that over time, individuals experiencing homelessness may develop greater resilience.  Impact of Social Support and Functioning: The results indicated a positive association between resilience and social support and functioning. Higher scores in social support and social functioning were related to higher resilience. Conversely, lower perceived stress was associated with better social support, social functioning, and more days of stable housing. This underscores the importance of social networks in managing stress and bolstering resilience.  Stable Housing and Stress: The study also noted that while resilience was not directly associated with the percentage of days stably housed, lower perceived stress was. This suggests that stable housing may be more directly effective in reducing stress rather than increasing resilience.  Implications for Interventions: Based on these findings, the study suggests that interventions for the homeless should not only focus on providing stable housing but also on building strong social support systems. These dual efforts can help mitigate the negative impacts of stress and contribute to better overall well-being. | Longitudinal descriptive study | High/Strong |
| Ellis et al. | 2022 | Interdisciplinary Approach to Community Resilience (CR): The development of the CR model was comprehensive, incorporating literature from a range of fields including medicine, psychology, and disaster preparedness. This suggests that understanding and improving community resilience requires a multidisciplinary perspective that considers a wide array of factors from neurobiology to public policy.  System Dynamics Modeling for Public Health: The application of system dynamics modeling indicates a sophisticated approach to understanding the complex interactions between public systems and policies. This modeling serves as a tool to visualize and quantify how different sectors like housing, education, law enforcement, and criminal justice interact and influence community health outcomes.  Impact of Structural Racism on Community Resilience: The main outcomes of the CR model show that structural racism within public systems can significantly suppress community resilience by influencing the distribution of resources and opportunities. This finding emphasizes the need for public health strategies to address systemic inequalities as a part of community health improvement efforts.  Policy Implications and Actionable Steps: The results advocate for public health leaders to use the CR model to collaborate with various sectors to address inequities rooted in structural racism. By doing so, they can systematically tackle issues contributing to negative experiences in childhood and community life, thereby enhancing community resilience and equity. | Systematic Review | Moderate/Moderate |
| Embleton et al. | 2016 | Lack of Empirical Data - The text highlights a critical gap in empirical data regarding the reasons for street involvement of children and youth. Without this data, policies and interventions may not effectively address the underlying causes that lead to this situation.  Primary Reasons for Street Involvement - The systematic analysis revealed poverty as the most commonly reported reason for street involvement, with family conflict and abuse also being significant factors. These causes were prevalent regardless of the level of human development or geographic region, indicating a universal need for targeted social support.  Impact of Socioeconomic Factors - The findings underscore the impact of socioeconomic inequities as a primary driver for children and youth becoming street-involved. The data suggest that addressing these inequities is essential for preventing street involvement and requires a concerted effort from governments and policymakers worldwide.  Delinquency a Minor Factor - Contrary to some perceptions, delinquency was infrequently cited as a reason for street involvement, which challenges the stereotype that street-connected children and youth are primarily involved in criminal activities. This insight is crucial for shaping public perception and policy to be more compassionate and supportive rather than punitive. | Systematic Review/Meta-analysis | High/Strong |
| Finfgeld-Connett | 2010 | Challenging Youth Circumstances: The study emphasizes that homeless women often face difficult situations in their youth, which impact their ability to prevent and resolve homelessness when they become adults. It suggests a connection between early life challenges and later difficulties in achieving stable housing.  Iterative Resolution Stages: Homelessness resolution is identified as a process occurring in stages, specifically crisis, assessment, and sustained action. This implies that resolving homelessness is not a single event but a series of steps that need to be addressed sequentially.  Role of Nurses and Empowerment: Nurses are recognized as key figures in supporting homeless women. They are encouraged to foster empowerment, aligning their approach with the Transtheoretical Model (which assesses an individual's readiness to act on a new healthier behavior) and the Harm Reduction Model (which seeks to reduce the negative consequences of certain behaviors). This insight suggests that nurses can be instrumental in facilitating progress through the stages of homelessness resolution.  Valued Services: The study indicates that services most valued by homeless women include those addressing physical and mental health, as well as child care assistance. This points to the need for a holistic approach to social services, acknowledging the complex needs of homeless women, which go beyond mere shelter. | Qualitative Analysis | Moderate/Moderate |
| Foroughi et al. | 2022 | Creation of a Meta-Framework: The research addresses the confusion surrounding the variety of resilience models for health systems by developing a meta-framework. This unified framework aims to simplify understanding and operationalizing resilience in health systems, making it more accessible for researchers and policymakers.  Systematic Review and Data Analysis: The study methodically collated and reviewed existing literature on health system resilience, employing the Critical Interpretive Synthesis method. The systematic review was extensive, utilizing multiple databases and tools such as MMAT for quality appraisal and MAXQDA 10 for data analysis, indicating a rigorous and comprehensive approach.  Identification of Framework Dimensions: The result of this analysis was the identification of five main themes that serve as dimensions for the framework. These include the phases of health system resilience, its attributes, the tools and strategies for resilience, and the foundational building blocks and goals of health systems. These dimensions offer a structured lens for analyzing resilience in health systems.  Operationalization and Policy Solutions: The discussion highlights the meta-framework's systemic and comprehensive nature, which not only allows for the assessment of resilience through various phases and objectives but also suggests policy solutions. The framework encourages the use of absorptive, adaptive, and transformative strategies as tools to achieve resilience in health systems. This aspect is particularly crucial as it translates theoretical understanding into practical steps for policymakers. | Qualitative Meta-Analysis | High/Strong |
| Funk et al. | 2022 | Higher Mortality Rates for the Homeless: The review indicates that individuals who are homeless have a higher rate of premature death compared to those who are not homeless. This highlights the severe impact that the state of homelessness has on an individual’s health and longevity.  Diverse Causes of Death: There is a variation in the causes of death among different subpopulations within the homeless community, such as veterans, families, youth, and the unsheltered. While causes of death have changed over time, the top ones identified include diseases like cancer (neoplasms), heart disease, and deaths related to substance use disorder.  Variability in Methodology and Findings: The studies reviewed used various methodologies and data sources, leading to a variation in findings. This suggests that there is a need for standardization in research to better understand and address the issue of mortality in the homeless population.  Need for Focused Research and Policy Action: The review underscores the necessity for research that focuses on subpopulation variations, policy implications, and the influence of mortality risk factors such as poverty and racism. This implies that tackling the issue of homelessness and its associated mortality rate requires a multifaceted approach that includes both research and policy reforms. | Systematic Review | Moderate/Moderate |
| Gartland et al. | 2019 | Variability in Measurement: There is a significant variability in how child resilience is measured, with most studies not using psychometrically validated tools. Only two studies out of 30 used a resilience measure for children, which highlights a gap in reliable assessment tools for this demographic.  Factors Influencing Resilience: A variety of factors contribute to resilience in children facing social adversity, including cognitive skills, emotion regulation, relationships with caregivers, and academic engagement. These factors were identified across different studies as being instrumental in fostering resilient outcomes.  Predominance of Individual Factors: While different domains such as child, family, school, and community factors were explored, individual factors were the most commonly investigated. This could indicate a research bias or a reflection of the importance of individual traits in resilience.  Intervention Strategies: The insights gained from the systematic review suggest potential starting points for public health interventions. These interventions could be aimed at enhancing cognitive and emotional skills, strengthening caregiver relationships, and promoting academic involvement to support children in overcoming the effects of social adversity. | Systematic Review | Moderate/Moderate |
| Grattan et al. | 2022 | Interplay of Risk and Resilience Factors: The experience of homelessness in young people is shown to impact their development in multiple dimensions, including social, emotional, and physical. This multi-faceted impact underscores the importance of understanding both risk and resilience factors—variables that can either contribute to or mitigate the likelihood of becoming homeless.  Systematic Review for Informed Interventions: A systematic review of existing research was conducted, which is critical to developing informed interventions. By analyzing 16 peer-reviewed quantitative studies, the authors were able to identify common risk and resilience factors. This structured approach to review literature ensures that interventions are based on evidence rather than assumption.  Development of a Provisional Model: The research findings were integrated into a developmental model of youth homelessness risk. This model offers a valuable framework for understanding how various factors contribute to homelessness and can be used to guide the creation of preventive services and strategies.  Clinical and Preventive Implications: The study identifies specific factors that are influential in the risk of youth homelessness, such as family difficulties, mental health, substance use, and educational background. The findings not only have clinical implications for service development but also highlight the potential for preventive strategies that could be implemented before homelessness occurs. | Systematic Review | High/Strong |
| Han et al. | 2023 | Significance of PCEs in Mitigating Childhood Adversity: The research indicates that positive childhood experiences have a modest inverse relationship with levels of childhood adversity. This suggests that while PCEs don't completely negate the impact of adverse experiences, they play a significant role in providing a counterbalance that can lead to more favorable outcomes in adulthood.  PCEs as Direct Promotive Factors: The majority of the studies reviewed found that PCEs often have a direct, promotive effect on adult outcomes across various domains such as mental health, psychosocial functioning, and physical health. This implies that PCEs themselves are valuable in fostering resilience and well-being in adults, independent of their role in mitigating adversity.  Need for Further Research in Diverse Contexts: Despite the growing body of literature, the review underscores the need for more research on PCEs in diverse and international samples. This points to the current gap in understanding how PCEs function across different cultural and socioeconomic backgrounds and indicates the importance of expanding research to these areas to build a more comprehensive understanding of PCEs' impact. | Systematic Review | Moderate/Moderate |
| Heaslip & Green et al. | 2021 | Health and Mortality Disparity: The background establishes that people experiencing homelessness face a significant risk of premature death. This underscores the need for robust health and social care services to bridge the gap in health disparities.  Challenges in Locating Services: The objective of the study highlights the difficulty homeless individuals have in identifying and locating appropriate health and social care services. This suggests that while services may exist, there are barriers to accessing information about them.  Barriers to Access: The results point out that despite the high prevalence of poor health among the homeless population, access to healthcare and wellbeing services is fraught with systemic, individual, and cultural hurdles. This indicates that the current support infrastructure, which relies heavily on third-sector charity organizations, may not be sufficiently integrated or proactive in reaching out to those in need.  Reliance on Informal Networks: The qualitative data emphasizes that homeless individuals often depend on word-of-mouth to access services, which can be unreliable and indicative of a lack of formal, accessible information channels.  Need for Systemic Review: The conclusion calls for a review of how information on health and wellbeing services is disseminated in local communities. It suggests that a systemic change is necessary to make information more accessible and to improve the health outcomes for the homeless population. | Mixed-Method Quantitative/Qualitative | Moderate/Moderate |
| Heaslip & Richer, et al. | 2021 | Health Inequality in Homelessness: The background highlights a critical issue — people who are homeless often face worse health outcomes than the general population. This inequality is exacerbated by difficulties in accessing healthcare services, pointing to the need for innovative solutions to improve health equity.  Technology as an Enabler: The expansion of technology offers a promising avenue to improve health outcomes for socially excluded groups, including homeless populations. The potential of technology, especially mobile health (mHealth) applications, to provide better access to health services is recognized, yet under-researched, prompting this review.  Research Gaps and Methodology: The methods section indicates a systematic approach to identify relevant studies, using an integrative review methodology and rigorous screening criteria. This approach addresses the lack of research by examining the available literature on the use of mHealth technologies by homeless populations and their impact on health.  Potential and Challenges of mHealth: The conclusion synthesizes the findings, acknowledging the potential of technology to support the health of homeless individuals. However, it also flags significant obstacles such as internet connectivity, data privacy, and trust. These challenges must be navigated to fully harness the benefits of health technology for this vulnerable population. The call for further research suggests a path forward to overcome these barriers and optimize mHealth solutions for the homeless. | Quasi-experimental | Moderate/Moderate |
| Heathcote et al. | 2019 | Positive Impact on RTW: The meta-analysis shows that resilience rehabilitation programs significantly increase the odds of individuals returning to work after an injury. The data indicates that participants in such programs are more than twice as likely to return to work compared to those who received standard care.  Reduction in RTW Time: Programs focused on resilience not only positively influenced the likelihood of returning to work but also reduced the time taken for individuals to RTW. This suggests that resilience programs may expedite the recovery process.  Increase in Self-Efficacy: There is a significant increase in self-efficacy scores among those who participate in resilience rehabilitation programs, indicating that these programs are effective in boosting the participants’ confidence in their ability to cope with challenges.  Importance of Workplace Support: The research identifies workplace support as a critical factor for favorable RTW outcomes, highlighting the importance of the work environment in the rehabilitation process. Moreover, it points out that individuals with musculoskeletal or orthopedic injuries benefit significantly from resilience programs. | Meta-Analysis | High/Strong |
| Hudson et al. | 2016 | Complex Challenges: Homeless individuals face unique obstacles in accessing palliative care due to chaotic lifestyles, stigma in mainstream health settings, and the significant strain on staff within hostels where end-of-life support may be provided. These challenges are multifaceted, including immediate day-to-day survival needs that compete with seeking health care and systemic inflexibilities that do not accommodate the unique situations of homeless individuals.  Systemic Inflexibility: Mainstream healthcare systems often lack the necessary flexibility to provide effective palliative care to homeless people. This inflexibility can manifest in rigid policies, insufficient training for healthcare professionals in dealing with the complexities of homelessness, and a lack of collaboration between different services that could otherwise contribute to more holistic and accessible care for this marginalized population.  Improvement Strategies: To improve palliative care access and provision for the homeless, the review suggests building trust between healthcare professionals and homeless individuals, fostering greater collaboration and flexibility across services, and enhancing training and support for all professionals involved. These strategies are aimed at creating a more responsive and equitable healthcare system that recognizes and adapts to the specific needs of homeless populations. | Qualitative Meta-Analysis | High/Strong |
| Karadzhov et al. | 2020 | Understanding Coping Strategies: Recognizing how individuals cope with SMI is critical for improving their quality of life and recovery. The paper adds to existing knowledge by providing a detailed interpretive review of coping with SMI during homelessness, highlighting the complex and dynamic coping processes affected by homelessness.  Impact of Homelessness on Coping: Homelessness can severely disrupt the ability to cope with life stressors and chronic mental illness due to stress, social isolation, and negative self-beliefs. However, despite severe social disadvantages, some individuals show resilience, positive adaptation, and personal growth.  Implications for Nursing Practice: The practice should be informed by the understanding that coping efforts are influenced by various adverse factors such as poverty and discrimination. Nurses should aim to support coping behaviors and help create conditions that facilitate personal growth and constructive meaning-making.  Qualitative Metasynthesis Findings: A qualitative metasynthesis based on first-person accounts revealed the intricate nature of coping strategies and the influence of contextual factors. It emphasizes that coping while facing homelessness and SMI is not uniform but varies greatly, with individuals drawing on internal and external resources to manage their circumstances. Nursing practice should adopt an approach that addresses the complex needs of those facing multiple disadvantages, focusing on strength and meaning. | Qualitative Meta-Analysis | High/Strong |
| Kennedy et al. | 2022 | Dynamic Social Network Change: The background highlights the dynamic nature of social networks for individuals transitioning out of homelessness, especially when engaged with harm reduction housing programs. It notes that social networks are not static and that changes include severing ties with street-based contacts, reconnecting with old contacts, and forming new connections, some of which may pose a risk of substance use.  Intervention Strategy - MI-SNI: The methods describe the pilot randomized controlled trial of the Motivational Interviewing-Social Network Intervention (MI-SNI), which is innovative in its approach to combine network visualization with Motivational Interviewing. This technique is used to provide feedback and motivate positive changes in residents’ social environments and behaviors.  Effective Outcomes: The results show significant differences between the MI-SNI group and the usual care group. Those who received MI-SNI had a greater reduction in network members who influenced alcohol or other drug (AOD) use and made healthier adjustments in their social networks over a three-month period.  Implications for Future Research: The conclusion provides an encouraging perspective on the potential of MI-SNI, suggesting that it may be beneficial for individuals experiencing homelessness and AOD issues to restructure their social networks positively. The promising results from this pilot study point towards the necessity for a larger-scale RCT to further test and verify the efficacy of the MI-SNI approach. | Experimental-Randomized/Control | High/Strong |
| Kirst et al. | 2014 | Hope as a Crucial Element: The study affirms that hope plays a vital role in the recovery process for homeless populations. It suggests that having stable housing can significantly boost individuals' optimism about their recovery journey.  Housing's Impact on Recovery Goals: The narratives from the participants illustrate that not only is housing seen as a critical stepping stone in their path to recovery, but it also aids in the clarification and visualization of their recovery goals. This implies that housing contributes to a more structured and goal-oriented recovery process.  Challenges with Housing Transition: Despite housing being generally beneficial, some participants encountered difficulties adjusting to their new living situations. Concerns about social isolation were highlighted, indicating that merely providing housing is not a panacea; social integration and support are also important.  Recommendations for Housing First Interventions: The conclusions suggest that interventions like Housing First should not only focus on providing housing but also integrate approaches that inspire hope and assist with the emotional and social challenges of adjusting to new housing. This holistic approach might enhance the effectiveness of the intervention in sustaining hope and promoting recovery. | Qualitative Analysis | Moderate |
| Larkin et al. | 2012 | Comprehensive Approach to Trauma and Adversity: The RIS model is recognized as a holistic method for addressing trauma and adversity. Unlike approaches that focus on a single aspect of an individual's experience or health, RIS encompasses a whole person perspective. This means it takes into account various elements of an individual's life and experiences, aiming to address not just the symptoms but the root causes of their challenges.  Link Between Childhood Trauma and Adult Issues: The ACE Study by the CDC and Kaiser Permanente underscores a significant connection between childhood trauma and long-term health and social issues in adulthood. This finding is crucial because it highlights the importance of early intervention and the need for comprehensive approaches like RIS to mitigate these long-term effects.  Practical Application in Social Services: The case study of the Committee on the Shelterless (COTS) in Petaluma, California, serves as a practical example of the RIS model in action. By employing RIS, COTS demonstrates how social service agencies can effectively use this approach to break cycles of homelessness, a problem often linked to ACEs. This showcases the model's applicability in real-world settings and its potential impact on challenging social issues.  Mobilizing Resilience Through Social Affiliations: A key component of the RIS model, as implemented by COTS, is the emphasis on building resilience through social affiliations. This aspect of the program underscores the importance of community and social support in the recovery process, suggesting that fostering strong social networks can be a crucial element in helping individuals overcome the impacts of childhood trauma.  Recommendation for Wider Implementation and Research: The authors advocate for the broader implementation of the RIS model in programs serving populations with ACE backgrounds, coupled with more research. This recommendation implies a belief in the effectiveness of the RIS model and a need for further empirical evidence to support its widespread adoption in various social service settings. | Descriptive report | Moderate/Moderate |
| Leonard | 1991 | Shift from Vulnerability to Resilience: The section suggests a paradigm shift in research perspective, moving away from viewing siblings of chronically ill children primarily as vulnerable. Instead, it advocates for focusing on their resilience and the factors that contribute to successful adaptation.  Reframing Research Questions: There's an emphasis on the need to rethink the questions being asked in research studies. Historically, studies have focused on the risks and challenges faced by siblings of chronically ill children. The section suggests that research should now concentrate on understanding the dynamics that enable these siblings to live successfully.  Focus on Successful Living: The section underscores the point that many children are indeed living successfully with disabled siblings. Recognizing and studying these success stories can lead to better interventions that support not just the siblings but also the families as a whole.  Identifying Risk and Protective Factors: While acknowledging the chronicity of the sibling's condition as a given, the section points out the importance of identifying both risk and protective factors. This can help in creating strategies to mitigate negative outcomes for able-bodied siblings, disabled children, and their parents. | Literature Review | Moderate/Moderate |
| Llistosella et al. | 2022 | Evidence-based Protective Factors: The integrative systematic review focused on identifying protective factors that contribute to resilience in young individuals at risk. By analyzing a large number of peer-reviewed articles, the review has uncovered over 60 protective factors that are statistically significant in helping children, adolescents, and young adults cope with challenges like violence, trauma, or socio-economic instability.  New Model Development: The research resulted in the creation of the Individual and Environmental Resilience Model (IERM). This new conceptual model distinguishes itself by incorporating a wide range of protective factors across ten domains and two dimensions of resilience, namely Individual Skills and Environmental.  Comprehensive Approach: The IERM is presented as a more comprehensive model compared to existing ones. By including a broad spectrum of domains—ranging from biological to emotional in the individual skills dimension and family to community in the environmental dimension—it provides a holistic framework for understanding resilience.  Implications for Interventions: The insights from the IERM are poised to inform the development of interventions. With its detailed classification of protective factors, the model may serve as a valuable tool for practitioners designing programs to foster resilience in at-risk youth, potentially leading to more tailored and effective resilience-promoting strategies. | Systematic Review | High/Strong |
| Liu et al. | 2020 | Transition Complexity: The transition from hospital to home is recognized as a critical and complex phase for elderly chronic patients due to the long-term and recurring nature of their conditions. The frequent travel for various levels of care highlights the need for special attention to this vulnerable population.  Impact of Transition Practices: Unhealthy transition practices can lead to adverse outcomes and increased hospital readmission rates. This underscores the importance of effective care transition protocols and practices to ensure patient safety and reduce the risk of negative health events.  Global Importance: The safe and high-quality transition of care is of global concern, with healthcare providers playing a pivotal role in facilitating a smooth transition for the elderly. This responsibility involves understanding and addressing the unique needs of older adults in transition.  Multifaceted Perspectives: The study aims to consider a wide range of perspectives, including those of older patients, caregivers, and healthcare providers. This comprehensive view is crucial in identifying what shapes health transitions and ensuring that interventions are well-informed and effective.  Facilitators and Inhibitors: The research results indicate that there are both facilitators and inhibitors affecting the transition process, categorized into themes of resilience, relationships and connections, and care transfer supply chain. Understanding these factors is key to developing targeted interventions to improve transitions.  Informing Interventions: The findings of the study are intended to inform the development of interventions that bolster resilience among older adults, foster strong relationships and connections, and ensure an uninterrupted supply chain for care transfers. These interventions aim to make the transition from hospital to home as seamless and safe as possible. | Quantitative Meta-Analysis | High/Strong |
| Mabhala et al. | 2017 | Complexity of Homelessness: The study underscores a shift from viewing homelessness merely as a lack of permanent housing to a more complex social and public health issue. This complexity includes the broader social contexts and conditions that contribute to homelessness, which traditional housing-focused interventions may not adequately address.  Importance of Personal Stories: By examining the personal narratives of homeless individuals, the research highlights the value of understanding homelessness from the perspective of those experiencing it. This person-centered approach offers insights into the social factors and conditions that precede and perpetuate homelessness.  Process and Resilience: The findings suggest that homelessness is not an abrupt event but a gradual process marked by the diminishing ability to cope with life's adversities. This process often culminates in the breakdown of significant relationships, emphasizing the role of resilience and social support in preventing homelessness.  Behavioural and Socio-Economic Factors: The study indicates that while individuals may attribute their homelessness to immediate behaviours such as substance abuse or conflicts with authorities, these are situated within a broader context of adverse social and economic conditions that they believe have limited their life opportunities, such as quality social connections, education, and stable employment. | Qualitative Analysis | Moderate/Moderate |
| Mao et al. | 2022 | Resilience Diversity: The research indicates that resilience among rescue workers is not uniform. While some rescuers experience significant physical and psychological impact from their work in disaster scenarios, others demonstrate resilience, or the ability to cope with adversity without negative consequences.  Characteristics of Resilience: The review identifies six domains that characterize the resilience of rescue workers: demographic and physical characteristics, personality traits, coping strategies, perceived resources, being equipped with special skills for disaster rescue, and having less adverse consequences from exposure to disaster.  Research and Application: The findings have practical implications for researchers and disaster managers. Understanding these resilience characteristics can help in developing frameworks to assess rescue workers' resilience and establish interventions aimed at improving their psychological wellbeing post-disaster.  Systematic Review for Comprehension: The study employs a systematic literature review, analyzing data from seven electronic databases and conducting hand searches to explore the resilience of rescuers comprehensively. This methodological approach allows for a thorough understanding of what constitutes 'positive resilience' among rescue workers. | Systematic Review | Moderate/Moderate |
| Marshall et al. | 2020 | Systemic Challenges: The research reveals that service providers and organizational leaders recognize that the existing systems often inhibit the thriving of persons who have experienced homelessness. They indicate a systemic issue where the focus is on housing as an end goal rather than a step towards greater stability and well-being, encapsulated in the sentiment that providing a house is seen as the solution to homelessness, without sufficient support for what comes next.  The Role of Community and Peer Support: There's an identified need for better community integration and the inclusion of peer expertise. This suggests that for individuals to thrive post-homelessness, they need to be part of a supportive community. The involvement of peers—those with lived experience of homelessness—can be particularly valuable in designing interventions that are both practical and empathetic.  Flexibility and Choice in Housing and Services: The study emphasizes the importance of providing options in housing and services, suggesting a move away from one-size-fits-all solutions. Individuals who have experienced homelessness should have the agency to choose the type of support and housing that best fits their needs, which is crucial for their ability to thrive. | Systematic Review/Meta-Analysis | High/Strong |
| Martins | 2008 | Resource Compromise and Health: The research highlighted that the lack of essential resources among homeless individuals directly compromises their health, indicating a vicious cycle where homelessness exacerbates health issues, and poor health can further entrench homelessness.  Deferred Care Leading to Crisis: A pattern of deferring health care until it becomes a crisis was identified. This suggests that homeless individuals may often wait until their health issues become severe before seeking help, which can lead to worse health outcomes and more complicated treatments.  Barriers to Health Care: Homeless individuals face multiple barriers to receiving health care. These include social triage, where they are prioritized lower than others; stigmatization and labeling; a disorganized system of care for the homeless; disrespect from health care providers; and a sense of invisibility within the health care setting.  Underground Resourcefulness: Despite these barriers, homeless individuals exhibit a significant level of resourcefulness. They develop and use underground strategies to cope with and navigate the challenges of being marginalized by both society and the health care system. | Qualitative Analysis | Moderate/Moderate |
| Mayar et al. | 2022 | Reiteration of Established Concepts: The review suggests that a significant portion of the resilience literature is not entirely novel but instead revisits established ideas from the domain of engineering systems stability. This implies that the concept of resilience, while being discussed as a new paradigm in various fields, often parallels older stability principles already present in engineering.  Systems Theory as a Comparative Framework: By using modern control systems theory as a lens, the paper positions all types of systems (across disciplines) in a unified framework based on inputs, state, and outputs. This approach underscores the universal applicability of control systems theory to understand resilience across different fields, emphasizing its cohesiveness and broad relevance.  Resilience as Adaptive Capacity: The review highlights that resilience is closely tied to the idea of adaptation in systems. Resilient systems are characterized by their ability to absorb disruptions and perturbations, either by returning to an original state or by transitioning to a new, suitable state. This capacity is facilitated by both passive and active feedback mechanisms within the system, which allow it to respond dynamically to changes. | Systematic Review (Engineering) | Moderate/Moderate |
| Mejia-Lancheros et al. | 2021 | Resilience as a Positive Influence: The study highlights that among homeless adults with mental illness, there is a positive longitudinal relationship between resilience levels and quality of life outcomes. This suggests that resilience may serve as a protective factor that could mitigate the negative impacts of homelessness on overall well-being.  Consistent Resilience Scores Over Time: Data showed that resilience scores remained relatively stable over the 6-year period across various data collection points. This consistency in resilience suggests it is a trait that can potentially be measured reliably over time among adults facing homelessness and mental health issues.  Need for Targeted Interventions: The findings underscore the importance of developing interventions that focus on building resilience. Given the association between higher resilience and better quality of life and mental health outcomes, there is a clear indication that services aiming to enhance resilience could be beneficial in improving the lives of the homeless population, particularly those with mental illnesses. | Descriptive-Secondary Analysis | Moderate/Moderate |
| Mitchell et al. | 2023 | Health and Mortality Risks: The systematic review indicated that people experiencing homelessness have notably poorer physical and mental health compared to the general population. They face a higher risk of hospitalisation, increased usage of emergency departments, higher mortality rates, and are more vulnerable to severe outcomes from illnesses like COVID-19.  Socioeconomic Consequences: The studies highlighted that homelessness is associated with several adverse socioeconomic outcomes. Individuals experiencing homelessness are more likely to face unemployment and incarceration. These factors suggest a cyclical relationship between homelessness and societal participation, where being homeless can lead to difficulties in gaining employment, which in turn may perpetuate the state of homelessness.  Importance of Early Intervention: The findings underscore that the experience of homelessness in childhood has particularly profound effects. This suggests that early-life interventions might be crucial in preventing the long-term consequences of homelessness and indicates a strong link between early adverse experiences and later health and social challenges. | Systematic Review | High/Moderate |
| Nevard et al. | 2021 | Vulnerable Children's Social Networks: The research highlights that vulnerable children and young people, excluding those from minority ethnic backgrounds, often have limited social networks. These impoverished networks can negatively impact their health and well-being. However, for those in minority ethnic groups, the research seems to indicate a different pattern that is not associated with impoverished networks.  Protective Role of Networks: Access to social networks acts as a protective factor for vulnerable children, mitigating negative outcomes. Immediate family members, in particular, provide crucial personal resources. This suggests that strengthening familial ties could be a key strategy in supporting vulnerable young populations.  Substitutability of Network Ties: For children and young people whose social networks are restricted, the research suggests that network ties can be substituted to some extent. This substitutability indicates that interventions can be designed to provide alternative forms of support when traditional network ties, such as family, are not available or sufficient. | Mixed Qualitative/Quantitative systematic Analysis | High/Strong |
| Nilsson et al. | 2019 | Adverse Life Events as Predictors: The research underscores adverse life events as significant risk factors for becoming homeless. Physical abuse and foster care experiences notably increase the likelihood of an individual becoming homeless, with odds ratios indicating a two to threefold increase in risk compared to those without such experiences.  Incarceration and Psychiatric Problems: A history of incarceration and suicide attempts are highlighted as strong predictors for homelessness. Psychiatric issues, particularly drug use problems, are also associated with a higher risk of homelessness. These factors suggest a correlation between homelessness and systemic as well as mental health challenges.  Sociodemographic Factors: The study suggests that certain sociodemographic factors, such as being female and having a partner, are associated with higher chances of exiting homelessness. Conversely, relationship problems, psychotic disorders, and drug use problems tend to reduce the likelihood of exiting homelessness.  Substantial Heterogeneity in the Data: The research notes high levels of heterogeneity (I2 > 90%) in most analyses, implying that the predictors of homelessness can vary significantly across different studies and populations. This suggests that while the identified factors are relevant, they may not apply uniformly across all contexts. | Systematic Review | Moderate/Moderate |
| Noh & Choi | 2020 | Recognition of a Gap in Intervention Studies: There's an acknowledgment that existing intervention studies rarely consider the family contexts of runaway adolescents, which may be critical in addressing their mental health needs effectively.  Use of a Structured Approach for Intervention Development: The Intervention Mapping protocol is utilized, which is a systematic approach to program planning and decision-making. This includes a literature review and interviews to understand the problem, setting behavioral and environmental outcomes, identifying determinants, and selecting methods to bring about change.  Combination of Individual and Family Approaches: The intervention designed is family-based and includes both individual and family therapeutic approaches. This suggests an understanding that addressing the issue requires looking beyond the individual adolescent to the family system they are a part of.  Implementation and Evaluation Strategy: It is planned that mental health nurses in community centers will implement the program, ensuring professional delivery. Moreover, a randomized controlled trial is proposed to rigorously evaluate the program's effectiveness, indicating a commitment to evidence-based practice. | Research Protocol Development with Literature Review | Moderate/Moderate |
| Onapa et al. | 2022 | Prevalence of Homelessness and Inadequate Housing: The section starts by highlighting the extensive nature of homelessness and housing inadequacy worldwide, with an estimated 150 million people being homeless and around 1.8 billion lacking adequate housing. This sets a global context for the importance of the issue.  Correlation Between Housing and Health: Although there is a widely accepted view that housing is a crucial determinant of health and can be a strategy to address health disparities among the homeless, there is still a lack of clear understanding and comprehensive development of this concept. The review aims to delve deeper into this correlation by analyzing intervention studies that focus on the health impacts of housing for the homeless.  Findings of the Systematic Review: The review included an analysis of previous reviews and studies focusing on permanent supportive housing interventions. These interventions often target homeless individuals with mental illnesses and provide them with affordable housing coupled with support services. The review found that while there are inconsistencies and issues in the literature, there is evidence to suggest that housing can, at least in the short term, improve certain health outcomes for homeless populations, particularly those suffering from HIV, anxiety, and depression. Despite the mixed results, the trend indicates a positive impact of housing on physical and mental health, well-being, and quality of life. | Systematic Review | High/Strong |
| Paul et al. | 2018 | Recognition of Personal Strengths: The study highlights that individuals from ethnoracial backgrounds who are facing homelessness and mental illness in Toronto recognize their personal strengths and attitudes as critical resources. These include hope, optimism, self-esteem, confidence, and spiritual beliefs. This recognition suggests that despite their compounded challenges, these individuals retain a sense of agency and internal resources that aid in their resilience.  Coping Strategies and Support Networks: Participants reported employing various coping strategies. Notably, they seek support from a network of family, friends, and professionals. This emphasizes the importance of a support system and social connections in managing their circumstances. Moreover, engaging in meaningful activities and socializing with peers serves as a way to navigate their daily struggles.  Shared Resilience with the Broader Homeless Population: The findings indicate that the coping mechanisms and sources of resilience used by ethnoracial individuals with mental illness and homelessness are akin to those of the general homeless population. This suggests that while the challenges faced may be compounded by racial discrimination, the fundamental human responses to adversity may remain consistent across different subpopulations of the homeless.  Implications for Services: The study concludes that there is a need for services to acknowledge and support the unique coping strategies and resilience sources employed by homeless individuals. Services should foster hope and provide recognition, which can be integral in the journey towards overcoming the complex challenges faced by this subpopulation. It also underscores the necessity for culturally competent and responsive services that can address the specific needs of ethnoracially diverse individuals. | Qualitative Analysis | High/Strong |
| Slockers et al. | 2018 | Background: We aimed to assess the contribution of specific causes-of-death to excess mortality of homeless persons and to identify differences in cause-specific mortality rates after vs. before implementing social policy measures.  Methods: We conducted a register based 10-year follow-up study of homeless adults in Rotterdam and calculated the proportion of deaths by cause-of-death in this cohort in the period 2001-2010. We estimated cause-specific mortality among the homeless compared to the general population with Standardized Mortality Ratios. We calculated Hazard Ratios adjusted for age and sex to compare mortality rates by cause-of-death among the homeless in the period after (2006-2010) vs. before (2001-2005) implementing social policy measures.  Results: Our cohort consisted of 2130 homeless persons with a mean age of 40, 3 years. Unnatural death, cardiovascular disease and cancer were the main causes of death. Compared to the general population of Rotterdam, the homeless had an excess risk of death for all causes. The largest mortality differences with Rotterdam citizens were observed for unnatural death (SMR 14.8, CI 11.5-18.7), infectious diseases (SMR 10.0, CI 5.2-17.5) and psychiatric disorders (SMR 7.7, CI 4.0-13.5). Mortality due to intentional injuries (suicide and homicide) differed significantly between the two study periods (HR 0.45, CI 0.20-0.97).  Conclusions: Reducing unnatural death should be a target in social policies aimed at improving the health of the homeless. We generated the hypothesis that social policies aimed at housing, work and improved contact with health care could be accompanied by less suicides and homicides within this vulnerable group. | Quasi-Experimental | High/Moderate |
| Tippens et al. | 2023 | Recent Increase in Attention: There has been a growing focus on the mental and psychosocial health of older refugees, with the majority of research published in the last decade. This indicates an increasing recognition of the importance of this issue within the research community.  Research-Location Discrepancy: There is a disparity between the geographical areas where most refugees reside (low- and middle-income countries) and where most mental health and psychosocial support (MHPSS) research is conducted. This suggests that there may be a lack of data and insights into the psychosocial resilience of older refugees in the regions where they are most in need.  Varied Determinants of Resilience: The determinants of psychosocial resilience among older refugees are highly varied and are influenced by the politico-historical context of migration, sociocultural backgrounds, and the specific postmigration needs, resources, and settings. This underscores the complexity of addressing mental health in this population.  Multisystemic Resilience Factors: Protective factors and resilience processes operate at multiple systemic levels. Macrosystem factors include security and access to services, mesosystem factors revolve around social support networks, and microsystem factors involve individual attributes like language skills and optimism. This indicates that interventions to enhance psychosocial resilience should be multidimensional and interdisciplinary, taking into account the various ecosystems that interact to affect older refugees' well-being. | Systematic Review | High/Strong |
| Tryon & Radzin | 1972 | Existential Vacuum and Mental Health: Viktor Frankl's theory posits that a lack of meaning and purpose in life leads to a state of existential vacuum, characterized by feelings of emptiness, boredom, and hopelessness. This condition is seen as a common human experience rather than a pathology. Studies have supported the connection between purpose in life and mental health, suggesting that having a clear sense of meaning is integral to psychological well-being.  Purpose-in-Life Test and Construct Validity: The Purpose-in-Life Test, operationalized from Frankl's concepts by Crumbaugh and Maholick, aims to measure an individual's sense of meaning and has been used to explore its relationship with mental health. In the study, additional measures like Block's Ego Resiliency Scale and Rokeach's Dogmatism Scale were used alongside biographical variables to assess the construct validity of the Purpose-in-Life Test.  Correlations with Ego Resiliency and Dogmatism: The study found that individuals with higher scores on the Purpose-in-Life Test tended to have higher ego resiliency and lower dogmatism. Ego resiliency relates to an individual's ability to adapt to changing circumstances while maintaining a sense of self, and dogmatism refers to the rigidity of an individual's beliefs. This suggests that those who have a stronger sense of purpose are more adaptable and less dogmatic.  Biographical Factors and Purpose in Life: There were significant positive correlations between an individual's purpose in life and their certainty of college major, certainty of future occupation, and courting status. This indicates that a sense of purpose is linked with having clear academic and professional goals and a commitment in personal relationships.  The study extends the understanding of how a sense of purpose in life, a central tenet of Frankl's logotherapy, is associated with various psychological and biographical factors, reinforcing its importance to mental health. | Quasi-Experimental Descriptive | Moderate/Strong |
| Tsai & Rosenheck | 2015 | Substance Use and Mental Health as Primary Risk Factors: The systematic review underscores substance use disorders and mental illness as the most significant and consistent risk factors for homelessness among US veterans. This finding suggests that interventions addressing these issues could be central to preventing and reducing veteran homelessness.  Socioeconomic Challenges: The review highlights low income and income-related factors as critical risk factors for homelessness. This insight suggests that economic support and interventions could play a vital role in supporting at-risk veterans.  Need for Comprehensive Research: The call for more studies that simultaneously address premilitary, military, and postmilitary risk factors indicates a gap in the current research. Understanding the interplay of these factors could lead to more effective, holistic approaches to preventing homelessness among veterans. | Systematic Review | High/Strong |
| Wyman et al. | 1992 | Caregiver Relationships: SR children reported having more positive relationships with their primary caregivers compared to SA children. This suggests that a strong, supportive bond with caregivers is crucial for resilience in the face of major life stress.  Family Environment Stability: SR children experienced more stable family environments. This insight underscores the importance of a stable home life for children's ability to cope with stress.  Discipline Practices: Inductive (reasoning-based) and consistent discipline within the family were more common among SR children. This points to the role of structured and understandable discipline in fostering resilience.  Future Expectations: SR children had more positive expectations for their future, indicating that a hopeful outlook might contribute to resilience. | Mixed-Method Quantitative/Qualitative | Moderate/Moderate |
| Zhang et al. | 2021 | Protective Role of Social Problem-Solving: The study highlights the importance of social problem-solving as a protective factor for homeless youth against suicidal ideation. By fostering better coping responses in stressful situations, it can reduce feelings of burdensomeness and thwarted belongingness, which are linked to suicidal thoughts.  Effectiveness of Cognitive Therapy for Suicide Prevention (CTSP): The research provides evidence that CTSP, when combined with Treatment as Usual (TAU), can significantly enhance the protective role of social problem-solving. This indicates that CTSP is effective in mitigating factors that contribute to suicidal ideation in homeless youth.  Role of Perceived Burdensomeness: The findings suggest that perceived burdensomeness is a mediating factor between social problem-solving skills and suicidal ideation. Specifically, CTSP helps to alleviate perceived burdensomeness, thereby reducing suicidal ideation among homeless youth.  Implications for Suicide Prevention Interventions: The study's results have broader implications for suicide prevention efforts, suggesting that interventions should focus on enhancing social problem-solving abilities to reduce suicide risk factors. This could lead to more effective support for high-risk groups such as homeless youth. | Experimental: Randomized Control Study | High/Moderate |
